# Supplementary material for: Identification of Clonality through Genomic Profile Analysis in Multiple Lung Cancers
Source: J Clin Med. 2020 Feb 20;9(2):573. doi: 10.3390/jcm9020573 (PMC7074554; doi:10.3390/jcm9020573)
Supplement: Supplementary file 1 [file jcm-09-00573-s001.zip › jcm-690359-supplementary/jcm-690359-supplementary.docx]

**Supplementary Table 1.** The genes targeted in the cancer panel.

| **No** | **Gene symbol** | **Chromosome** | **Number of Amplicons** | **Total Bases** | **Covered Bases** | **Overall Coverage** |
| --- | --- | --- | --- | --- | --- | --- |
| 1 | AKT1 | chr14 | 26 | 1573 | 1497 | 95% |
| 2 | AKT2 | chr19 | 27 | 1576 | 1543 | 98% |
| 3 | AKT3 | chr1 | 30 | 1624 | 1624 | 100% |
| 4 | ARID1A | chr1 | 76 | 7058 | 6023 | 85% |
| 5 | ARID1B | chr6 | 75 | 6950 | 5965 | 86% |
| 6 | ARID2 | chr12 | 71 | 5718 | 5643 | 99% |
| 7 | ASCL4 | chr12 | 5 | 532 | 382 | 72% |
| 8 | ATM | chr11 | 147 | 9791 | 9439 | 96% |
| 9 | BRAF | chr7 | 37 | 2481 | 2224 | 90% |
| 10 | CDKN2A | chr9 | 9 | 962 | 612 | 64% |
| 11 | COBL | chr7 | 48 | 4151 | 3977 | 96% |
| 12 | CREBBP | chr16 | 96 | 7639 | 7071 | 93% |
| 13 | CTNNB1 | chr3 | 32 | 2486 | 2486 | 100% |
| 14 | CUL3 | chr2 | 42 | 2561 | 2495 | 97% |
| 15 | EGFR | chr7 | 60 | 4189 | 4135 | 99% |
| 16 | EP300 | chr22 | 90 | 7555 | 7182 | 95% |
| 17 | EPHA7 | chr6 | 44 | 3175 | 3154 | 99% |
| 18 | ERBB2 | chr17 | 57 | 4080 | 3808 | 93% |
| 19 | ERBB3 | chr12 | 59 | 4440 | 4374 | 99% |
| 20 | FGFR1 | chr8 | 41 | 2825 | 2816 | 100% |
| 21 | FGFR2 | chr10 | 43 | 2910 | 2842 | 98% |
| 22 | FGFR3 | chr4 | 34 | 2752 | 2215 | 81% |
| 23 | FOXP2 | chr7 | 36 | 2487 | 2469 | 99% |
| 24 | HRAS | chr11 | 11 | 683 | 683 | 100% |
| 25 | KEAP1 | chr19 | 24 | 1925 | 1845 | 96% |
| 26 | KMT2D | chr12 | 192 | 17154 | 15854 | 92% |
| 27 | KRAS | chr12 | 10 | 737 | 681 | 92% |
| 28 | MAP2K1 | chr15 | 18 | 1292 | 1239 | 96% |
| 29 | MET | chr7 | 59 | 4427 | 4396 | 99% |
| 30 | MGA | chr15 | 110 | 9428 | 9345 | 99% |
| 31 | MLL | chr11 | 144 | 12279 | 11875 | 97% |
| 32 | NF1 | chr17 | 136 | 9161 | 9023 | 99% |
| 33 | NFE2L2 | chr2 | 23 | 1868 | 1826 | 98% |
| 34 | NOTCH1 | chr9 | 99 | 8008 | 7078 | 88% |
| 35 | NOTCH2 | chr1 | 101 | 7809 | 7539 | 97% |
| 36 | NRAS | chr1 | 9 | 610 | 610 | 100% |
| 37 | PIK3CA | chr3 | 50 | 3407 | 3282 | 96% |
| 38 | PTEN | chr10 | 18 | 1302 | 1223 | 94% |
| 39 | RASA1 | chr5 | 55 | 3412 | 3216 | 94% |
| 40 | RB1 | chr13 | 55 | 3057 | 2902 | 95% |
| 41 | RBM10 | chrX | 48 | 3228 | 3079 | 95% |
| 42 | RIT1 | chr1 | 13 | 771 | 771 | 100% |
| 43 | SETD2 | chr3 | 91 | 7905 | 7663 | 97% |
| 44 | SLIT2 | chr4 | 76 | 4972 | 4854 | 98% |
| 45 | SMAD4 | chr18 | 24 | 1769 | 1715 | 97% |
| 46 | SMARCA4 | chr19 | 74 | 5399 | 5055 | 94% |
| 47 | SOX2 | chr3 | 9 | 964 | 883 | 92% |
| 48 | STK11 | chr19 | 23 | 1392 | 1343 | 97% |
| 49 | TP53 | chr17 | 22 | 1383 | 1351 | 98% |
| 50 | TP63 | chr3 | 34 | 2360 | 2227 | 94% |
| 51 | TSC1 | chr9 | 49 | 3705 | 3603 | 97% |
| 52 | TSC2 | chr16 | 92 | 5834 | 5677 | 97% |
| 53 | U2AF1 | chr21 | 15 | 880 | 870 | 99% |

**Supplementary Table 2.** Patient characteristics

|  | **Age** | **Sex** | **Smoking habit** | **Tumor location** | **Occurence of tumors** | **Operative procedure** | **Size(mm)** | **p-TNM** | **p-stage** | **Histopathology** |
| --- | --- | --- | --- | --- | --- | --- | --- | --- | --- | --- |
| 1 | 73 | F | Non-smoker | rS9, rS4 | Synchronous | right lower lobectomy,  right middle wedge | 11, 8 | ①T1bN0M0 ②T1miN0M0 | IA | ① inv muc Ad, ly0, v0, pl0 ② MIA, ly0, v0, pl0 |
| 2 | 74 | M | Smoker | rS2, rS3, | Synchronous | right upper lobectomy | 35, 35 | ①T2aN0M0 ②T2aN0M0 | IB | ① inv Ad, solid, ly1, v0, pl3 ② inv Ad, papillary, ly0, v1, pl0 |
| 3 | 67 | M | Smoker | rS6, rS3 | Synchronous | right upper lobectomy,  S6 segmentectomy | 15, 20 | ①T2aN0M0 ②T1bN0M0 | IB | ① inv Ad, solid, ly0, v0, pl1 ② inv Ad, lepidic, ly0, v0, pl0 |
| 4 | 57 | M | Smoker | rS6, rS1 | Synchronous | right lower lobectomy,  right upper wedge | 60, 15 | ①T3N1M0 ②T2aN0M0 | IIIA | ① Sq, ly1, v1, pl1 ② Sq, ly0, v1, pl2 |
| 5 | 54 | M | Smoker | rS1, leftS1+2 | Synchronous | right upper lobectomy,  left upper segmentectomy | 32,7 | ①T2aN0M0 ②T1miN0M0 | IB | ① inv Ad, lepidic, ly1, v1, pl0 ② MIA, ly0, v0, pl0 |
| 6 | 82 | M | Smoker | rS6, rS10 | Synchronous | right lower lobectomy | 20, 25 | ①T1bN0M0 ②T1aN0M0 | IA | ① inv Ad, lepidic, ly0, v0, pl0 ② inv Ad, lepidic, ly0, v0, pl0 |
| 7 | 72 | M | Smoker | leftS1+2, leftS1+2 | Synchronous | left upper segmentectomy | 10, 15 | ①T1miN0M0 ②TisN0M0 | IA | ① MIA, ly0, v0, pl0 ② AIS, ly0, v0, pl0 |
| 8 | 73 | M | Smoker | leftS8, rS2 | Synchronous | left lower lobectomy,  right upper wedge | 18, 20 | ①T2aN0M0 ②T1cN0M0 | IB | ① small, ly0, v1, pl2 ② inv Ad, papillary, ly0, v0, pl0 |
| 9 | 67 | F | Non-smoker | rS3, leftS9 | Synchronous | right upper lobectomy,  left lower lobectomy | 20, 25 | ①T1bN0M0 ②T2aN0M0 | IB | ① inv Ad, lepidic, ly0, v0, pl0 ② inv Ad, papillary, ly0, v0, pl1 |
| 10 | 59 | F | Non-smoker | rS1, rS6 | Metachronous | right upper lobectomy,  right lower resection | 25, 14 | T2aN0M0 | IB | inv Ad, lepidic, ly0, v1, pl1 |
| 11 | 73 | M | Smoker | leftS3, leftS8 | Synchronous | left pneumonectomy | 33, 8 | ①T2aN0M0 ②TisN0M0 | IB | ① Sq, ly0, v1, pl0 ② AIS, ly0, v0, pl0 |
| 12 | 77 | M | Smoker | leftS10, leftS10 | Synchronous | left lower lobectomy | 52, 20 | ①T3N1M0 ②T2aN0M0 | IIIA | ① Sq, ly1, v1, pl1 ② small, ly0, v1, pl2 |
| 13 | 78 | M | Smoker | rS3, rS1 | Synchronous | left upper lobectomy | 15, 17 | ①T1bN0M0 ②TimiN0M0 | IA | ① inv Ad, acinar, ly0, v1, pl0 ② MIA, ly0, v0, pl0 |
| 14 | 79 | M | Smoker | leftS8, leftS6 | Synchronous | left lower lobectomy | 27, 13 | ①T1cN0M0 ②TisN0M0 | IA | 1. Sq, ly0, v0, pl0 2. AIS, ly0, v0, pl0 |
| 15 | 58 | M | Smoker | leftS1+2, leftS1+2 | Metachronous | left upper wedge,  left upper wedge | 25, 12 | T2aN0M0 | IB | ① inv Ad, acinar, ly0, v0, pl1 ② inv Ad, solid, ly0, v1, pl1 |
| 16 | 85 | M | Non-smoker | rS4, rS7 | Synchronous | right middle lobectomy,  right lower wedge | 18, 6 | ①T1bN0M0 ②T1miN0M0 | IA | ① inv Ad, acinar, ly0, v0, pl0 ② MIA, ly0, v0, pl0 |
| 17 | 65 | M | Smoker | rS2, rS4 | Synchronous | right upper lobectomy,  right middle wedge | 35, 7 | ①T2aN0M0 ②T1aN0M0 | IB | ① inv Ad, lepidic, ly1, v0, pl0 ② inv Ad, papillary, ly0, v0, pl0 |
| 18 | 74 | M | Non-smoker | leftS3, leftS3 | Synchronous | left upper lobectomy | 60, 20 | ①T3N0M0 ②T1bN0M0 | IIB | ① Sq, ly0, v1, pl3 ② Sq, ly0, v0, pl0 |
| 19 | 67 | F | Non-smoker | rS8, leftS3 | Synchronous | right lower lobectomy,  left upper lobectomy | 33, 50 | ①T2aN1M0 ②T1aN0M0 | IIB | ① inv Ad, acinar, ly1, v1, pl0 ② inv Ad, lepidic, ly0, v0, pl0 |
| 20 | 69 | M | Smoker | rS8, leftS6 | Synchronous | right lower lobectomy,  left lower lobectomy | 10, 70 | ①T1aN0M0 ②T1aN0M0 | IA | ① Sq, ly0, v0, pl0 ② inv Ad, lepidic, ly0, v0, pl0 |
| 21 | 75 | M | Smoker | rS1, leftS10 | Metachronous | right upper lobectomy,  left lower wedge | 32, 18 | ①T2aN0M0 ②T2aN0M0 | IB | ① Sq, ly1, v1, pl3 ② inv Ad, acinar, ly0, v0, pl1 |
| 22 | 74 | M | Smoker | rS9, rS3 | Metachronous | right lower lobectomy,  right upper wedge | 10, 15 | ①T1aN0M0 ②T1bN0M0 | IA | ① inv muc Ad, ly0, v0, pl0 ② small, ly0, v1, pl0 |
| 23 | 73 | M | Smoker | rS2, leftS1+2 | Metachronous | right upper lobectomy,  left upper wedge | 55, 20 | T3N0M0 | IIB | Sq, ly1, v1, pl0 |
| 24 | 63 | F | Smoker | rS10, leftS8 | Metachronous | right lower lobectomy,  left lower lobectomy | 35, 50 | ①T2aN0M0 ②T3N1M0 | IIIA | ① inv Ad, acinar, ly0, v1, pl0 ② inv Ad, solid, ly1, v2, pl3 |
| 25 | 63 | M | Smoker | rS9-10, leftS6 | Metachronous | right lower lobectomy,  left S6 segmentectomy | 80, 10 | T4N0M0 | IIIA | inv muc Ad, ly0, v0, pl0 |
| 26 | 72 | M | Smoker | rS9, rS6 | Synchronous | right lower lobectomy | 40, 10 | ①T2aN2M0 ②T1aN0M0 | IIIA | ① Sq, ly0, v1, pl0 ② Sq, ly0, v1, pl0 |
| 27 | 75 | M | Smoker | rS1, leftS1+2 | Synchronous | right upper lobectomy,  left upper segmentectomy | 30, 20 | ①T2aN0M0 ②T1miN0M0 | IB | ① inv Ad, solid, ly0, v1, pl1 ② MIA, ly0, v0, pl0 |
| 28 | 70 | M | Smoker | leftS3, rS2 | Metachronous | left upper lobectomy,  right upper wedge | 40, 15 | ①T2aN0M0 ②TNM0 | IB | ① inv Ad, papillary, ly1, v0, pl0 ② inv Ad, papillary, ly0, v0, pl0 |
| 29 | 80 | M | Smoker | rS4, rS6 | Synchronous | right middle lobectomy,  right lower resection | 25, 12 | ①T1cN0M0 ②T1bN0M0 | IA | ① Pleomorphic, ly0, v0, pl0 ② inv Ad, acinar, ly0, v0, pl0 |
| 30 | 74 | M | Smoker | rS1, rS1 | Synchronous | right upper lobectomy | 30, 20 | ①T1cN2M0 ②T2aN0M0 | IIIA | ① Adenosq, ly1, v1, pl0 ② inv Ad, papillary, ly0, v0, pl1 |
| 31 | 73 | M | Smoker | rS1, rS6 | Synchronous | right upper lobectomy,  right lower wedge | 30, 8 | ①T2aN0M0 ②T1aN0M0 | IB | ① Sq, ly0, v1, pl1 ② Sq, ly0, v0, pl0 |
| 32 | 74 | F | Non-smoker | rS9, leftS9 | Metachronous | right lower lobectomy,  left lower wedge | 32, 10 | T2aN0M0 | IB | inv Ad, micropapillary, ly3, v1, pl0 |
| 33 | 62 | M | Smoker | left1+2 x 3 | Synchronous | left upper lobectomy | 15, 14, 12 | T3N0M0 | IIB | inv Ad, solid, ly1, v1, pl1 |
| 34 | 77 | M | Smoker | leftS10, rS5 | Metachronous | left lower lobectomy,  right middle wedge | 35, 15 | ①T2aN0M0 ②T2aN0M0 | IB | ① Sq, ly0, v0, pl2 ② LCNEC, ly0, v1, pl1 |
| 35 | 72 | M | Smoker | leftS4, rS1+rS1 | Synchronous | left upper lobectomy,  right upper lobectomy | 10, 7, 2 | ①T1aN0M0 ②T1aN0M0 ③T1aN0M0 | IA | ① inv Ad, papillary, ly0, v0, pl0 ② LCNEC, ly0, v0, pl0 ③ inv Ad, solid, ly0, v0, pl0 |
| 36 | 56 | M | Smoker | leftS8, leftS1+2, leftS10 | Synchronous | left lower lobectomy,  left upper wedge | 45, 26, 25 | ①T2bN0M0 ②T2aN0M0 ③T1cNM0 | IIA | ① inv muc Ad, ly0, v0, pl0 ② Sq, ly0, v0, pl2 ③ Sq, ly0, v0, pl0 |
| 37 | 74 | M | Smoker | rS9, leftS3 | Metachronous | ight lower lobectomy,  left upper lobectomy | 50, 21 | T2bN0M0 | IIA | inv muc Ad, ly0, v1, pl0 |

p-TNM, pathological-tumor-node-metastasis; M, male; F, female; S, segment; r, right; Sq, squamous cell carcinoma; Ad, adenocarcinoma; inv, invasive; muc, mucinous; MIA, microinvasive adenocarcinoma; AIS, adenocarcinoma in situ, wedge; wedge resection; adenosq; adenosquamous carcinoma; LCNEC, large cell neuroendocrine carcinoma
