# Supplementary material for: Identification of Clonality through Genomic Profile Analysis in Multiple Lung Cancers
Source: J Clin Med. 2020 Feb 20;9(2):573. doi: 10.3390/jcm9020573 (PMC7074554; doi:10.3390/jcm9020573)
Supplement: Supplementary file 1 [file jcm-09-00573-s001.zip › jcm-690359-supplementary/Supple Table 3-JCM.pdf]

Supplemental Table 3. Mutation data in each cancer sample

Case	Tumor location	Gene	Mutation	Position	Ref.	Tumor var.	AF
1	right S9	U2AF1	p.Ser34Phe	chr21:44524456	G	G/A	33%
		KRAS	p.Gly12Asp	chr12:25398280	GCCAC	GCCAC/GCCAT	32%
		KMT2D	p.Arg2645Ter	chr12:49433620	G	G/A	6%
		SOX2-OT|SOX2	p.Met294Ile	chr3:181431030	G	G/A	4%
	right S4	EGFR	p.E746-R748 del	chr7:55242465			31%
2	right S2	TP53	splice site	chr17:7577157	T	T/A	76%
		EP300	p.Pro2322Ser	chr22:41574679	C	C/T	7%
	right S3	CDKN2A	splice site	chr9:21970900	C	C/T	95%
		NF1	p.Glu2358fs	chr17:29670037	AAGTAT	A/A	95%
		TP53	splice site	chr17:7578556	T	T/C	85%
		CREBBP	p.Tyr1705Ser	chr16:3781251	T	T/G	18%
		NF1	p.Thr700Ser	chr17:29553550	C	C/G	17%
		KMT2D	p.Asp632Glu	chr12:49445570	G	G/C	12%
		KMT2D	p.Gln3943Leu	chr12:49426659	CT	CT/AA	8%
		NF1	p.Arg720Trp	chr17:29553609	C	C/T	5%
		KMT2D	p.Asp4690Asn	chr12:49423191	C	C/T	4%
3	right S6	TP53	p.Cys238Phe	chr17:7577108	C	C/A	51%
		MAP2K1	p.Lys57Asn	chr15:66727455	G	G/T	39%
		FOXP2	p.Gln250Lys	chr7:114271658	C	C/A	31%
		ATM	p.Ser601Asn	chr11:108122758	G	G/A	9%
		NF1	p.Pro2159Ser	chr17:29664433	C	C/T	8%
		SMAD4	p.Thr197Ala	chr18:48581285	A	A/G	8%
		EP300	p.Pro2314Thr	chr22:41574655	C	C/A	6%
		KMT2A	p.Ala3862Asp	chr11:118392074	C	C/A	5%
		NOTCH1	p.Cys1890Tyr	chr9:139395269	C	C/T	5%
	right S3	KRAS	p.Gly12Ala	chr12:25398284	C	C/G	58%
		ARID1B	p.Gly927Ter	chr6:157469985	G	G/T	15%
		MGA	p.Thr245Ala	chr15:41961825	A	A/G	13%
		SMAD4	p.Pro320Ser	chr18:48591795	C	C/T	12%
		COBL	p.Glu502Lys	chr7:51098509	C	C/T	11%
		KMT2D	p.Leu3920Gln	chr12:49426729	A	A/T	8%
		TSC2	p.Asp1465Tyr	chr16:2134616	G	G/T	8%
		RBM10	p.Glu184Asp	chrX:47030582	G	G/T	6%
		ERBB2	p.Pro856Thr	chr17:37881374	C	C/A	6%
		NOTCH2	p.Gly156Arg	chr1:120539905	C	C/T	5%
		HRAS	p.Ala11Val	chr11:534291	G	G/A	4%
		CREBBP	p.Ser1912Leu	chr16:3779313	G	G/A	4%
4	right S6	NF1	p.Tyr2476Phe	chr17:29677306	A	A/T	50%
		NRAS	p.Gln61Lys	chr1:115256528	TTG	TTG/TTT	49%

		ARID2	p.Thr219Ser	chr12:46215221	C	C/G	45%
		NFE2L2	p.Asp178His	chr2:178097182	C	C/G	44%
		EP300	p.Ser2328fs	chr22:41574692	GTCCT	GTCCT/GTCCCT	41%
		KMT2D	p.Arg2830Ter	chr12:49432651	G	G/A	29%
	right S1	SMARCA4	p.Glu371Ter	chr19:11098593	G	G/T	75%
		TP53	p.Arg248Trp	chr17:7577528	GATGGGCCTCCGGTTC	GATGGGCCTCCGGTTC/GATGGGCCTCCAGTTC	74%
		FOXP2	p.Pro277Leu	chr7:114271740	C	C/T	64%
		RIT1	p.Thr70Ser	chr1:155880247	T	T/A	43%
		MGA	p.Asp339His	chr15:41962107	G	G/C	39%
		MGA	p.Glu1249Lys	chr15:42021449	G	G/A	35%
		NF1	p.Leu2125Val	chr17:29663878	C	C/G	8%
5	right S1	TP53	p.Arg280Thr	chr17:7577094	C	C/G	56%
		PTEN	p.Lys342fs	chr10:89720854	A	A/ATACTTTTCTCCAAATTT	15%
		SOX2-OT|SOX2	p.Met294Ile	chr3:181431030	G	G/A	8%
	left S1+2	RBM10	p.Glu721Ter	chrX:47044469	G	G/T	48%
		EGFR	p.Leu858Arg	chr7:55259514	CT	CT/CG	30%
6	right S6	MAP2K1	p.Ser222Thr	chr15:66774188	T	T/A	37%
	right S10	TP53	p.Val173Glu	chr17:7578368	A	A/T	54%
		KEAP1	p.Trp497Ter	chr19:10600364	C	C/T	4%
7	left S1+2	EGFR	p.E746-A750 del*	chr7:55242465	AGGAATTAAGAGAAGC	AGGAATTAAGAGAAGC/A	25%
	left S1+2	MET	p.Asp1028His	chr7:116412043	G	G/C	36%
8	left S8	TP53	p.Cys242Phe	chr17:7577553	ATGCAGGAACTGT	ATGCAGGAACTGT/ATGAAGGAACTGT	86%
		EP300	p.Lys1783Arg	chr22:41573063	A	A/G	48%
		NOTCH1|MIR4673	p.Glu286Ter|	chr9:139413904	C	C/A	47%
		EGFR	p.Phe481Leu	chr7:55227976	T	T/A	46%
		NOTCH1	p.Gln58Ter	chr9:139418400	G	G/A	45%
		NF1	p.Arg440Gln	chr17:29533316	G	G/A	30%
		PTEN	p.Thr277Ile	chr10:89720679	C	C/T	29%
	right S2	TP53	p.Arg249Ser	chr17:7577528	GATGGGCCTCCGGTTC	GATGGGCCTCCGGTTC/GATGGGACTCCGGTTC	84%
		RASA1	p.Gly434Ter	chr5:86649020	G	G/T	78%
		CDKN2A	p.Asp74Tyr	chr9:21971138	C	C/A	72%

		FGFR1	p.Lys436Glu	chr8:38277122	T	T/C	58%
		KMT2D	p.Leu4467His	chr12:49425088	A	A/T	4%
9	right S3	EGFR	p.Leu858Arg	chr7:55259515	TG	TG/GG	10%
		ATM	p.Glu1971Lys	chr11:108181035	G	G/A	7%
		KMT2D	p.Asp1749Glu	chr12:49437723	A	A/C	4%
	left S9	SOX2-OT|SOX2	|p.Glu282Val	chr3:181430993	A	A/T	4%
		KMT2D	p.Asp632Glu	chr12:49445570	G	G/C	4%
10	right S1	TP53	p.Ile156Thr	chr17:7578265	A	A/G	63%
		EGFR	p.Glu746_Ala750del	chr7:55242466	AGGAATTAAGAGAAGC	AGGAATTAAGAGAAGC/A	38%
		KEAP1	p.Arg234Trp	chr19:10602878	G	G/A	40%
		SETD2	p.Asn1384Lys	chr3:47161974	A	A/C	7%
		SMARCA4	p.Phe1059Ser	chr19:11136983	T	T/C	7%
		RBM10	p.Arg722Cys	chrX:47044472	C	C/T	5%
	right S6	TP53	p.Ile156Thr	chr17:7578265	A	A/G	56%
		EGFR	p.Glu746_Ala750del	chr7:55242466	AGGAATTAAGAGAAGC	AGGAATTAAGAGAAGC/A	49%
11	left S3	CDKN2A	p.Asp108Tyr	chr9:21971036	C	C/A	69%
		CDKN2A	p.Leu104fs	chr9:21971041	ACGTCCAGCCGCGCC	ACGTCCAGCCGCGCC/A	69%
		NOTCH1	p.Cys1490Trp	chr9:139399878	G	G/C	56%
		TP53	p.Arg280Ile	chr17:7577094	GGTCTCT	GGTCTCT/GGTCTAT	52%
		SMARCA4	p.Asp779Tyr	chr19:11123685	G	G/T	43%
	left S8	EGFR	p.Leu858Arg	chr7:55259515	TG	TG/GG	29%
12	left S10	TP53	p.Arg280Thr	chr17:7577094	GGTCTCT	GGTCTCT/GGTCTGT	80%
		NFE2L2	p.Leu30Phe	chr2:178098957	G	G/A	72%
		ERBB2	p.Asp1144His	chr17:37883959	G	G/C	4%
		CREBBP	p.Leu551Ile	chr16:3831230	G	G/T	4%
	left S10	KMT2D	p.Gln3969Leu	chr12:49426582	T	T/A	54%
		TP53	p.Val197Met	chr17:7578256	TCCACTCGGATAAGATGCTGAGGAGGGG	TCCACTCGGATAAGATGCTGAGGAGGGG/TCCATTCGGATAAGATGCTGAGGAGGGG	18%
		KMT2D	p.Cys778fs	chr12:49445134	A	A/AC	18%
		KMT2D	p.Asp632Glu	chr12:49445570	G	G/C	5%
	LN#12	TP53	p.Arg280Thr	chr17:7577094	GGTCTCTCC	GGTCTCTCC/GGTCTGTCC	74%
		NFE2L2	p.Leu30Phe	chr2:178098957	G	G/A	38%

		SOX2-OT|SOX2	|p.Met294Ile	chr3:181431030	G	G/A	7%
		SOX2-OT|SOX2	|p.Ser295Phe	chr3:181431032	C	C/T	6%
	LN#7	TP53	p.Arg280Thr	chr17:7577094	GGTCTCT CC	GGTCTCTCC/G GTCTGTCC	76%
		NFE2L2	p.Leu30Phe	chr2:178098957	G	G/A	39%
		SOX2-OT|SOX2	|p.Met294Ile	chr3:181431030	G	G/A	6%
		SOX2-OT|SOX2	|p.Ser295Phe	chr3:181431032	C	C/T	5%
		SOX2-OT|SOX2	|p.Gln296Ter	chr3:181431034	C	C/T	4%
13	right S3	EGFR	p.Leu858Arg	chr7:55259515	TG	TG/GG	33%
	right S1	RBM10	p.Tyr573Ter	chrX:47040994	C	C/G	43%
14	left S8	CDKN2A	splice site	chr9:21971208	C	C/A	72%
		TP53	p.Glu294Ter	chr17:7577047	GTGGTGA GGCTCCC CTTTCT	GTGGTGAGGC TCCCCTTTCT/G TGGTGAGGCT ACCCTTTCT	68%
		EPHA7	p.Arg107Met	chr6:94120731	C	C/A	45%
		ATM	p.Gly1746fs	chr15:41989202	T	T/G	38%
		MGA	p.Phe665Cys	chr10:123239501	T	T/C	32%
		FGFR2	p.Tyr780Cys	chr12:46243935	G	G/A	23%
		ARID2	p.Val677Met	chr1:243828144	A	A/C	16%
		AKT3	p.Phe72Val	chr1:243828146	G	G/T	14%
		AKT3	p.Thr71Lys	chr16:3779773	G	G/T	14%
		CREBBP	p.Pro1759Thr	chr12:46245717	T	T/A	14%
		ARID2	p.Cys1271Ser	chr11:108172431	CTGGACA T	CTGGACAT/C	13%
		SMARCA4	p.Phe1059Ser	chr19:11136983	T	T/C	13%
		ERBB3	p.Asp857Ala	chr12:56491678	A	A/C	12%
		KMT2D	p.Lys2032Arg	chr12:49435886	T	T/C	10%
		FGFR3	p.Cys484Trp	chr4:1807115	C	C/G	10%
		ARID2	p.Ala1275Asp	chr12:46245730	C	C/A	10%
		ARID1A	p.Asn472Ser	chr1:27057707	A	A/G	8%
		KMT2D	p.Glu4829Ala	chr12:49421821	T	T/G	8%
		ARID1A	p.His495Gln	chr1:27057777	T	T/G	6%
		KMT2A	p.Ser3339Ala	chr11:118376622	T	T/G	6%
		KMT2A	p.Met411Ile	chr11:118343107	G	G/A	6%
		AKT1	splice site	chr14:105239791	C	C/G	6%
		FGFR3	p.Lys422Gln	chr4:1806239	A	A/C	5%
		FGFR1	p.Val782Gly	chr8:38271476	A	A/C	4%
		COBL	p.Gln527His	chr7:51097212	C	C/A	4%
		SOX2-OT|SOX2	|p.Thr232Ser	chr3:181430843	C	C/G	4%
	left S6	RBM10	p.Glu254Ter	chrX:47034480	G	G/T	66%
		KMT2A	p.Glu856Ter	chr11:118344440	G	G/T	39%

		BRAF	p.Gly469Ala	chr7:140481402	CC	CC/GC	31%
		KMT2A	p.Met411Ile	chr11:118343107	G	G/A	8%
		MAP2K1	p.Ala283Glu	chr15:66777482	C	C/A	7%
		KMT2D	p.Glu4829Ala	chr12:49421821	T	T/G	6%
		EGFR	p.Glu829Lys	chr7:55259427	G	G/A	5%
		NF1	p.Met1271Lys	chr17:29562732	T	T/A	5%
		ARID2	p.Ala1275Asp	chr12:46245730	C	C/A	5%
		ARID1A	p.Asn472Ser	chr1:27057707	A	A/G	4%
		CTNNB1	p.Cys439Ter	chr3:41275151	C	C/A	4%
		KMT2D	p.Glu641Ala	chr12:49445544	T	T/G	4%
15	left S1+2	TSC2	p.Cys519Ser	chr16:2114384	T	T/A	42%
		COBL	p.Arg1158His	chr7:51094274	C	C/T	33%
		CDKN2A	p.Leu65del	chr9:21971163	GAGC	GAGC/G	19%
		FGFR3	p.Ala636Thr	chr4:1807841	G	G/A	17%
		SMARCA4	p.Trp284Cys	chr19:11097672	G	G/T	16%
		FGFR3	p.Cys484Trp	chr4:1807115	C	C/G	10%
		SMARCA4	p.Ala723Thr	chr19:11121100	G	G/A	9%
		TP53	p.Gln104Ter	chr17:7579377	G	G/A	7%
		ERBB3	p.Ile399Leu	chr12:56486781	A	A/C	7%
		AKT2	p.Lys181Glu	chr19:40747877	T	T/C	6%
		CREBBP	p.Tyr1705Cys	chr16:3781251	T	T/C	6%
		NOTCH1|MIR4673	p.Asn304Lys|	chr9:139413230	A	A/C	5%
		NOTCH1|MIR4673	p.Ala305Asp|	chr9:139413228	G	G/T	5%
		NOTCH1|MIR4673	p.Asn304His|	chr9:139413232	T	T/G	5%
	left S1+2	TSC2	p.Cys519Ser	chr16:2114384	T	T/A	71%
		CDKN2A	p.Leu65del	chr9:21971163	GAGC	GAGC/G	60%
		FGFR3	p.Ala636Thr	chr4:1807841	G	G/A	28%
		TP53	p.Gln104Ter	chr17:7579377	G	G/A	25%
		FGFR3	p.Lys422Gln	chr4:1806239	A	A/C	20%
		FGFR3	p.Cys484Trp	chr4:1807115	C	C/G	11%
		SMARCA4	p.Ala723Thr	chr19:11121100	G	G/A	8%
		ERBB3	p.Cys552Tyr	chr12:56487924	G	G/A	7%
		NFE2L2	p.Leu84Pro	chr2:178098794	A	A/G	6%
		MGA	p.Leu1660Pro	chr15:42035137	T	T/C	6%
		MGA	p.Ser2952Phe	chr15:42059135	C	C/T	6%
		FGFR2	p.Ser110Gly	chr10:123325000	T	T/C	4%
		EP300	p.Gly2139Asp	chr22:41574131	G	G/A	4%
		TP63	p.His443Tyr	chr3:189590762	C	C/T	3%
16	right S4	EGFR	p.Leu858Arg	chr7:55259515	T	T/G	26.2
		KMT2D	p.Ser633Leu	chr12:49445568	G	G/A	4.27

	right S7	EGFR|EGFR-AS1	p.Asp770_Asn771insAsnProHis|	chr7:55249010	G	G/GACAACCCCC	17.4
17	right S2	MET	p.Leu1330fs	chr7:116435940	GAT	GAT/G	10.27
	right S4	BRAF	p.Val600Glu	chr7:140453136	A	A/T	14.99
		TP53	p.Leu45Gln	chr17:7579553	A	A/T	14.2
18	left S3	TP53	p.Val157Phe	chr17:7578443	AGATGGCCATGGCGCGGACGCGGGTGC	AGATGGCCATGGCGCGGACGCGGGTGC/AGATGGCCATGGCGCGGAAGCGGGTGC	48%
		BRAF	p.Arg199Thr	chr7:140508704	C	C/G	29%
		TP63	p.Ser38Ala	chr3:189455578	T	T/G	12%
		SOX2-OT|SOX2	|p.Ser295Phe	chr3:181431032	C	C/T	9%
		SOX2-OT|SOX2	|p.Met294Ile	chr3:181431030	G	G/A	7%
		TSC2	p.Gln110Ter	chr16:2103445	C	C/T	5%
		NF1	p.Arg711Cys	chr17:29553582	C	C/T	3%
	left S3	KMT2D	p.Met3349Val	chr12:49431094	T	T/C	56%
		KMT2A	p.Pro2357Arg	chr11:118373677	C	C/G	41%
		STK11	p.Phe354Leu	chr19:1223125	C	C/G	39%
		COBL	p.Asp497Asn	chr7:51098524	C	C/T	21%
		KMT2A	p.Pro3243Thr	chr11:118376334	C	C/A	20%
		NOTCH2	p.Ala1901Val	chr1:120462014	G	G/A	18%
		ERBB2|MIR4728	p.Arg929Gln|	chr17:37882020	G	G/A	16%
		ARID2	p.Gln1403Ter	chr12:46246113	C	C/T	15%
		AKT3	p.Ala373Val	chr1:243716076	G	G/A	14%
		AKT3	p.Thr71Lys	chr1:243828146	G	G/T	14%
		AKT3	p.Pro326Thr	chr19:1223039	C	C/A	14%
		STK11	p.Tyr18Cys	chr1:243859012	T	T/C	14%
		EP300	p.Gln2191Ter	chr22:41574286	C	C/T	13%
		SETD2	p.Thr2103Ile	chr3:47098966	G	G/A	12%
		MET	p.Pro749Ser	chr7:116398655	C	C/T	12%
		CTNNB1	p.Gly1942Asp	chr15:42041630	G	G/A	11%
		MGA	p.Arg587Ter	chr3:41277290	C	C/T	11%
		ARID1B	p.Asp519Asn	chr6:157150373	G	G/A	10%
		NOTCH1	p.Cys489Tyr	chr11:108121658	G	G/A	10%
		ATM	p.Ala886Ser	chr11:108139154	G	G/T	10%
		ATM	p.Pro143Gln	chr9:139417616	G	G/T	10%
		ARID2	p.Ala707Val	chr12:46244026	C	C/T	10%
		CREBBP	p.Asp357Tyr	chr19:10602509	C	C/A	9%
		KEAP1	p.His96Arg	chr19:10610423	T	T/C	9%
		KEAP1	p.Ser2315Gly	chr16:3778105	T	T/C	9%
		NOTCH2	p.Pro1351Leu	chr11:118353176	C	C/T	8%

		NOTCH2	p.Ser1353Asn	chr15:42028520	G	G/A	8%
		TP63	p.Ala456Thr	chr17:29533363	G	G/A	8%
		ARID1B	p.Arg21Cys	chr3:189349365	C	C/T	8%
		FOXP2	p.Trp573Ter	chr7:114299725	G	G/A	8%
		MET	p.Cys783Tyr	chr1:120496183	C	C/T	8%
		NOTCH1	p.Gln767Arg	chr1:120496231	T	T/C	8%
		KMT2A	p.Pro4520Leu	chr12:49424788	G	G/A	8%
		KMT2D	p.Val1088Met	chr7:116415114	G	G/A	8%
		KMT2D	p.Val392Ile	chr9:139412670	C	C/T	8%
		KMT2D	p.Leu5326Ile	chr12:49418437	G	G/T	8%
		ERBB3	p.Thr114Met	chr12:108169333	C	C/T	8%
		ASCL4	p.Gly1094Ser	chr6:157502247	G	G/A	8%
		MGA	p.Ser717Ala	chr12:49445317	A	A/C	8%
		NF1	p.Gly776Arg	chr12:56490880	G	G/A	8%
		NOTCH2	p.Met518Ile	chr6:157150372	G	G/A	7%
		CUL3	p.Asp578Asn	chr13:49027165	G	G/A	7%
		CUL3	p.Ala1721Val	chr1:120464910	G	G/A	7%
		FGFR3	p.Glu1919Lys	chr15:42041560	G	G/A	7%
		ARID1B	p.Thr1938Met	chr22:41573528	C	C/T	7%
		FGFR1	p.Ala5339Val	chr12:49418397	G	G/A	7%
		FGFR1	p.Asp485Asn	chr2:225367714	C	C/T	7%
		NOTCH1	p.Pro215Ser	chr8:38285510	G	G/A	7%
		ATM	p.Glu851Lys	chr9:139405640	C	C/T	7%
		KMT2D	p.Asp828His	chr15:42002945	G	G/C	7%
		KMT2D	p.Glu458Gly	chr4:1806657	A	A/G	7%
		RB1	p.Met557Ile	chr11:108122627	G	G/A	7%
		MGA	p.Val4407Met	chr12:49425269	C	C/T	7%
		MGA	p.Pro566Leu	chr2:225362480	G	G/A	7%
	LN#4	TP53	p.Val157Phe	chr17:7578443	AGATGGC CATGGCG CGGACGC GGGTGC	AGATGGCCAT GGCGCGGAC GCGGGTGC/A GATGGCCATG GCGCGGAAGC GGGTGC	50%
		BRAF	p.Arg199Thr	chr7:140508704	C	C/G	28%
		TP63	p.Ser38Ala	chr3:189455578	T	T/G	5%
19	right S8	FOXP2	p.Glu740Gln	chr7:114329976	G	G/C	7%
		ARID1A	p.Ser993Phe	chr1:27093047	C	C/T	6%
		KMT2A	p.Glu2665Lys	chr11:118374600	G	G/A	6%
		CREBBP	p.Tyr1705Ser	chr16:3781251	T	T/G	5%
		KMT2D	p.Asp632Glu	chr12:49445570	G	G/C	4%
	left S3	EGFR	p.Leu858Arg	chr7:55259515	TG	TG/GG	32%
		RBM10	p.Lys729Ter	chrX:47044493	A	A/T	26%

20	right S8	KMT2D	p.Gln3892Ter	chr12:49426814	G	G/A	54%
		TP53	p.Pro190Leu	chr17:7578280	G	G/A	52%
		CREBBP	p.Arg1173Ter	chr16:3807902	G	G/A	40%
		KEAP1	p.Arg470Cys	chr19:10600447	G	G/A	24%
	left S6	EGFR	p.Leu858Arg	chr7:55259515	T	T/G	23%
		MGA	splice site	chr15:42057261	G	G/T	6%
		EGFR	p.Ala871Gly	chr7:55259554	C	C/G	3%
21	right S1	TP63	p.Ser38Ala	chr3:189455578	T	T/G	17%
	left S10	TP53	p.Glu294fs	chr11:108150250	G	G/T	33%
		ATM	p.Arg1106Met	chr12:49426508	G	G/A	23%
		KMT2D	p.Gln3994Ter	chr17:7577064	TC	TC/T	8%
		NF1	p.Pro268Ser	chr17:29509597	C	C/T	4%
22	right S9	STK11	p.Pro221Leu	chr19:1220644	C	C/T	37%
		NOTCH1	p.Glu794Ter	chr9:139407560	C	C/A	5%
	right S3	RB1	p.Gln217Ter	chr13:48934194	C	C/T	94%
		KMT2D	p.Arg321Leu	chr12:49446848	C	C/A	91%
		TP53	p.Gln192Ter	chr17:7578275	G	G/A	90%
		KMT2D	p.Gln3994Ter	chr12:49426508	G	G/A	5%
23	right S2	ARID1A	p.Met937fs	chr1:27092788	A	A/AT	58%
		TP53	p.Arg158_Ala159 delinsPro	chr17:7578454	GCGC	GCGC/G	54%
		ERBB2	p.Ser463Gly	chr17:37872066	A	A/G	32%
		NFE2L2	p.Arg34Leu	chr2:178098944	C	C/A	31%
		SLIT2	p.Cys1130fs	chr4:20598103	AT	AT/A	28%
		TP63	p.Ser38Ala	chr3:189455578	T	T/G	8%
		ATM	splice site	chr11:108204696	G	G/A	7%
	leftS1+2	ARID1A	p.Met937fs	chr1:27092788	A	A/AT	50%
		TP53	p.Arg158_Ala159 delinsPro	chr17:7578454	GCGC	GCGC/G	40%
		ERBB2	p.Ser463Gly	chr17:37872066	A	A/G	29%
		SLIT2	p.Cys1130fs	chr4:20598103	AT	AT/A	27%
		NFE2L2	p.Arg34Leu	chr2:178098944	C	C/A	22%
		SOX2-OT|SOX2	|p.Ser295Phe	chr3:181431032	C	C/T	6%
		TP63	p.Ser38Ala	chr3:189455578	T	T/G	5%
24	right S10	PIK3CA	p.Glu542Lys	chr3:178936082	G	G/G	24%
		SOX2-OT|SOX2	|p.Gln296Ter	chr3:181431034	C	C/C	6%
		SOX2-OT|SOX2	|p.Met294Ile	chr3:181431030	G	G/G	4%
	left S8	KRAS	p.Gly12Asp|p.Gly 12Asp	chr12:25398283	ACC	ACC/ACC	29%

		TP53	p.Val197Glu	chr17:7578256	TCCACTCGGATAAGATGCTGAGGAGGGG	TCCACTCGGATAAGATGCTGAGGAGGGG/TCCACTCGGATAAGATGCTGAGGAGGGG	26%
		MGA	p.Trp169Ter	chr15:41961598	G	G/G	23%
	LN#12	KRAS	p.Gly12Asp|p.Gly12Asp	chr12:25398283	ACC	ACC/ATC	24%
		TP53	p.Val197Glu	chr17:7578256	TCCACTCGGATAAGATGCTGAGGAGGGG	TCCACTCGGATAAGATGCTGAGGAGGGG/TCCTCTCGGATAAGATGCTGAGGAGGGG	21%
		MGA	p.Trp169Ter	chr15:41961598	G	G/A	19%
		NF1	p.Arg711Cys	chr17:29553582	C	C/T	6%
25	right S9	KRAS	p.Gly12Cys	chr12:25398280	GCCACC	GCCACC/GCCACA	71%
		EGFR	p.Ala16Val	chr7:55087017	C	C/T	23%
		ARID1A	p.Trp1844Ter	chr1:27105920	G	G/A	22%
		AKT1	p.Cys296Gly	chr14:105239659	A	A/C	8%
		FGFR3	p.Gly192Asp	chr4:1803223	G	G/A	6%
		KMT2D	p.Asp1749Glu	chr12:49437723	A	A/C	6%
		SMARCA4	p.Leu724Phe	chr19:11121103	C	C/T	4%
		TSC2	p.Ala1238Thr	chr16:2131697	G	G/A	4%
	left S6	KRAS	p.Gly12Cys|p.Gly12Cys	chr12:25398283	ACC	ACC/ACA	39%
		ARID1A	p.Trp1844Ter	chr1:27105920	G	G/A	15%
		KMT2D	p.Asp632Glu	chr12:49445570	G	G/C	7%
26	right S9	TP53	p.Asn239Asp	chr17:7577553	ATGCAGGAACTGTT	ATGCAGGAACTGTT/ATGCAGGAACTGTC	77%
		LINC00441|RB1	|p.Pro24fs	chr13:48878116	CG	CG/C	73%
		KMT2D	p.Arg2410Leu	chr12:49434324	C	C/A	50%
	right S6	TP53	p.Tyr220Cys	chr17:7578190	T	T/C	45%
		PTEN	p.Leu182Ter	chr10:89711927	T	T/A	23%
		TP63	p.Ser38Ala	chr3:189455578	T	T/G	23%
		NFE2L2	p.Gly81Ser	chr2:178098804	C	C/T	16%
		NFE2L2	p.Glu82Asp	chr2:178098799	T	T/A	13%
	LN#11	TP53	p.Asn239Asp	chr17:7577553	ATGCAGGAACTGTT	ATGCAGGAACTGTT/ATGCAGGAACTGTC	77%
		LINC00441|RB1	|p.Pro24fs	chr13:48878116	CG	CG/C	62%
		KMT2D	p.Arg2410Leu	chr12:49434324	C	C/A	31%

		SOX2-OT|SOX2	|p.Gln296Ter	chr3:181431034	C	C/T	6%
		SOX2-OT|SOX2	|p.Ser295Pro	chr3:181431031	T	T/C	5%
	LN#7	TP53	p.Tyr220Cys	chr17:7578190	T	T/C	48%
		PTEN	p.Leu182Ter	chr10:89711927	T	T/A	44%
		NFE2L2	p.Gly81Ser	chr2:178098804	C	C/T	38%
		TP63	p.Ser38Ala	chr3:189455578	T	T/G	17%
		NOTCH1	p.Met2363Val	chr9:139391104	T	T/C	7%
27	right S1	TP53	p.Arg248Trp|p.Arg248Trp	chr17:7577528	GATGGGCCTCCGGTTC	GATGGGCCTCCGGTTC/GATGGGCCTCCAGTTC	20%
		PTEN	splice site	chr10:89720648	TA	TA/T	17%
		KRAS	p.Gly12Cys|p.Gly12Cys	chr12:25398283	ACC	ACC/ACA	16%
		SETD2	p.Lys556Ter	chr3:47164460	T	T/A	16%
		BRAF	p.Val600Leu	chr7:140453133	TTCAC	TTCAC/TTCAA	15%
	left S1+2	TP53	splice site	chr17:7578370	C	C/A	12%
		KRAS	p.Gly12Cys	chr12:25398285	C	C/A	11%
		NF1	p.Gln959Arg	chr17:29556878	A	A/G	6%
28	left S3	RBM10	p.Trp723Ter	chrX:47044477	G	G/A	66%
		EGFR	p.Leu858Arg	chr7:55259515	T	T/G	48%
		EGFR|EGFR-AS1	p.Arg776Cys|	chr7:55249028	C	C/T	27%
	right S2	KRAS	p.Gln61His	chr12:25380275	T	T/A	16%
29	right S4	TP53	p.Ser94Ter	chr17:7579406	G	G/C	33%
		KMT2D	p.Lys1712fs	chr12:49438034	CCT	CCT/C	20%
		RASA1	p.Val544Ala	chr5:86665650	T	T/C	19%
		KMT2D	p.Arg688fs	chr12:49445405	T	T/TC	16%
		CREBBP	p.Gln2084Arg	chr16:3778797	T	T/C	16%
		KMT2D	p.Ala4206Thr	chr12:49425872	C	C/T	13%
		AKT2	p.Met136Val	chr19:40748476	T	T/C	11%
		SETD2	p.Gln1164Pro	chr3:47162635	T	T/G	8%
		ARID2	p.Ala838Val	chr12:46244419	C	C/T	7%
		RASA1	p.Phe246Leu	chr5:86628367	T	T/C	5%
		FOXP2	p.Gly371Val	chr7:114284787	G	G/T	5%
		ARID1B	p.Pro908Ser	chr6:157469928	C	C/T	4%
	right S6	TP53	p.Gly244Ser	chr17:7577551	C	C/T	34%
		KMT2D	p.Gln3994Ter	chr12:49426508	G	G/A	4%
		NF1	p.Pro268Ser	chr17:29509597	C	C/T	4%
30	right S1	NF1	p.Trp221Ter	chr17:29508734	TG	TG/T	31%
		CDKN2A	p.Pro70Arg	chr9:21971149	G	G/C	23%
		TP53	p.Glu298Ter	chr17:7577046	C	C/A	23%
		SFTPB	down stream	chr2:85883742	ATTAT	ATTAT/A	20%

		EP300	p.Gln149Leu	chr22:41513542	A	A/T	11%
	right S3	NF1	p.Trp221Ter	chr17:29508734	TG	TG/T	41%
		TP53	p.Glu298Ter	chr17:7577046	C	C/A	39%
		CDKN2A	p.Pro70Arg	chr9:21971149	G	G/C	35%
		SFTPB	down stream	chr2:85883742	ATTAT	ATTAT/A	22%
		EP300	p.Gln149Leu	chr22:41513542	A	A/T	19%
	LN#4R	NF1	p.Trp221Ter	chr17:29508734	TG	TG/T	66%
		TP53	p.Glu298Ter	chr17:7577046	C	C/A	33%
		CDKN2A	p.Pro70Arg	chr9:21971149	G	G/C	27%
		PIK3CA	p.His1047Leu	chr3:178952085	A	A/T	22%
		EP300	p.Gln149Leu	chr22:41513542	A	A/T	16%
31	right S1	CDKN2A	p.Asp84Val	chr9:21971107	T	T/A	59%
		TP53	splice site	chr17:7579311	C	C/A	52%
		BRAF	p.Gly469Ala	chr7:140481402	CC	CC/GC	38%
	right S6	TP53	p.Pro152Leu	chr17:7578475	G	G/A	44%
		PIK3CA	p.Gln731Arg	chr3:178941873	A	A/G	4%
32	right S9	KRAS	p.Gly12Val	chr12:25398284	C	C/A	45%
		TP53	p.Arg175Leu	chr17:7578406	C	C/A	18%
	left S9	KRAS	p.Gly12Val	chr12:25398284	C	C/A	64%
		TP53	p.Arg175Leu	chr17:7578406	C	C/A	42%
		FGFR3	p.Ala634Thr	chr4:1807841	G	G/A	12%
33	left S1+2	TP53	p.Thr256Lys	chr17:7577514	G	G/T	43%
		KMT2A	p.Pro1351Leu	chr11:118353176	C	C/T	35%
		CDKN2A	p.Met52fs	chr9:21971175	CTCCGCC ACTCGGG CGCTGCC CATCATCA	CTCCGCCACTC GGGCGCTGCC CATCATCA/C	32%
	left S1+2	TP53	p.Thr256Lys	chr17:7577514	G	G/T	27%
		KMT2A	p.Pro1351Leu	chr11:118353176	C	C/T	16%
	left S1+2	TP53	p.Thr256Lys	chr17:7577514	G	G/T	46%
		KMT2A	p.Pro1351Leu	chr11:118353176	C	C/T	22%
		SMARCA4	p.Glu708Asp	chr19:11121057	G	G/T	11%
34	left S10	NFE2L2	p.Leu30Phe	chr2:178098957	G	G/A	28%
		FGFR2	p.Asn637Ser	chr10:123247581	T	T/C	3%
	right S5	RB1	p.Gln850Ter	chr13:49050864	C	C/T	95%
	LN#7	RB1	p.Gln850Ter	chr13:49050864	C	C/T	92%
35	left S4	TSC1	p.Glu887Asp	chr9:135772962	C	C/A	26%
		ARID1B	p.Ala2063Ser	chr6:157528462	G	G/T	16%
		ARID2	p.Arg143Cys	chr12:46211461	C	C/T	14%
		FGFR3	p.Arg471Trp	chr4:1806695	C	C/T	10%
	right S1	STK11	p.Gly257Glu	chr19:1221247	G	G/A	93%
		RB1	p.Glu539Ter	chr13:48955499	G	G/T	83%
		TSC1	p.Phe154Leu	chr9:135798781	G	G/T	80%

		AKT3	p.Glu240Lys	chr1:243736329	C	C/T	63%
		NOTCH2	p.Pro527fs	chr1:120508178	GC	GC/G	47%
		NOTCH2	p.Gly272Val	chr1:120529642	C	C/A	43%
36	left S8	KRAS	p.Ala59Thr	chr12:25380283	C	C/T	21%
	left S1+2	SMARCA4	p.Asp1284Tyr	chr19:11144518	G	G/T	31%
		SLIT2	p.Arg144Gly	chr4:20469409	A	A/G	3%
	left S10	NFE2L2	p.Glu79Gln	chr2:178098810	C	C/G	33%
		CUL3	p.Glu375Gln	chr2:225370756	C	C/G	30%
		SFTPA1	down stream	chr10:81375527	G	G/T	17%
37	right S9	CTNNB1	p.Leu427Phe	chr3:41275113	C	C/T	72%
		TP53	p.Gly115Val	chr17:7578469	C	C/A	69%
		KMT2D	p.Pro3145Ser	chr12:49431706	G	G/A	27%
		AKT3	p.Trp410Cys	chr1:243708833	C	C/A	26%
		SETD2	p.Gln97Ter	chr3:47165837	G	G/A	22%
		NOTCH1	p.Glu181Lys	chr9:139417503	C	C/T	10%
		MGA	p.Arg700Ser	chr15:41991269	A	A/C	8%
		SETD2	p.Arg1744Gly	chr3:47129650	T	T/C	7%
		SMARCA4	p.Val1501Ala	chr19:11152218	T	T/C	7%
		KMT2A	p.Ser2255Leu	chr11:118373371	C	C/T	6%
		ERBB3	p.Ala1017Glu	chr12:56493734	C	C/A	5%
		NF1	p.Ser856Asn	chr17:29556200	G	G/A	5%
		KMT2D	p.Asp1749Glu	chr12:49437723	A	A/C	4%
	left S3	CTNNB1	p.Leu427Phe	chr3:41275113	C	C/T	64%
		TP53	p.Gly154Val	chr17:7578469	C	C/A	61%
		AKT3	p.Trp410Cys	chr1:243708833	C	C/A	42%
		KMT2D	p.Pro3145Ser	chr12:49431706	G	G/A	24%
		KMT2D	p.Gln3994Ter	chr12:49426508	G	G/A	3%
